# Mr. Pulley on a Case of Urinous Vomiting

**Published:** 1813-06

**Authors:** John Pulley


*To the Editors of the Medical and Physical Journal.*

GENTLEMEN,

ON reading the observations of Dr. Yeats on Ischuria in the last Number of your Journal, I was much surprised to find unnoticed a case of reputed urinous vomiting which occurred a year or two ago at Bedford, in the person of Ann Foulkes. This case excited much interest, and the opinion of Dr. Yeats stood opposed to the united opinion of several other medical gentlemen. The doctor, believing implicitly in the allegations of the woman and her friends, strongly supported her cause, and pledged himself to publish the history of her disease; for which purpose he narrowly investigated her case, and took copious notes. Mr. Gibbon, a surgeon of this town, and myself, differing totally from the doctor on the existence of the complaint, equally pledged ourselves to reply to his statement, and now rest only on our oars until he shall redeem his pledge. At this time, I abstain from all observation on the individual instance, and the topic of urinous vomiting in general; but when the proper time arrives, I shall hold myself bound to remark both on one and the other.

I am, Gentlemen,
Your obedient Servant,
JOHN PULLEY.

